# Microstimulation of Interstitial Nucleus of Cajal Evokes Directionally Disconjugate Eye Movements in Monkeys With Pattern Strabismus

**DOI:** 10.1167/iovs.63.12.6

**Published:** 2022-11-03

**Authors:** Adam Pallus, Mark M. G. Walton

**Affiliations:** 1 Washington National Primate Research Center, University of Washington, Seattle, Washington, United States

**Keywords:** neurophysiology, strabismus, exotropia, esotropia, interstitial nucleus of Cajal (INC)

## Abstract

**Purpose:**

Pattern strabismus is characterized by a horizontal misalignment of the eyes that varies with vertical eye position. This disorder has traditionally been described, and treated, as overaction or underaction of the oblique muscles. In recent years, evidence has accumulated that indicate that the disorder is associated with abnormal cross-talk between brainstem pathways that contribute to the horizontal and vertical components of eye movements. The present study was designed to investigate the hypothesis that the key abnormalities are at the level of, or downstream from, the interstitial nucleus of Cajal (INC).

**Methods:**

Microstimulation was applied to the INC in two mature rhesus monkeys with “A” pattern strabismus that was experimentally induced in infancy. We asked whether the evoked movements would be vertical and conjugate, as has been previously reported in normal monkeys, or would be directionally disconjugate (i.e. with oblique or horizontal movement observed for at least one eye).

**Results:**

Evoked movements were conjugate and vertical for a minority of sites but, for most sites, the evoked movements were directionally disconjugate. Moreover, there was typically a convergent change in horizontal strabismus when the evoked movements were upward and a divergent change when the evoked movements were downward.

**Conclusions:**

Microstimulation of INC in monkeys with A-pattern strabismus evokes movements with the expected directional disconjugacies, implying that the key neural abnormalities are within, or downstream from, this structure. High site-to-site variability in the conjugacy/disconjugacy of evoked movements rules out the hypothesis that the abnormalities are solely peripheral.

In pattern strabismus, there is a correlation between the horizontal strabismus angle and vertical eye position. An “A” pattern, for example, is characterized by increased exotropia, or decreased esotropia, on downgaze.^1^^,^^2^ These abnormalities are often associated with abnormal torsion, an observation that led to the idea that the disorder may be attributable to over- or underaction of the oblique muscles. The development of non-human primate models^3^ of infantile strabismus has allowed the disorder to be investigated at the neurophysiological level.^4^^–^^18^ This work has provided compelling evidence that the cross-axis disconjugacies that characterize pattern strabismus are associated with abnormalities in the brainstem circuits that drive eye movements (for 2 recent review articles that discuss the relevant evidence in detail, see Refs. 19 and 20). Several authors have suggested that the cross-axis disconjugacies that characterize pattern strabismus result, in part, from abnormal cross-talk between brainstem pathways that normally encode the horizontal and vertical components of eye movements.^9^^,^^10^^,^^13^^,^^17^^,^^21^^–^^23^

Although there is compelling evidence to support this general idea, very little is known about what form this cross-talk might take, or where in the brain it might occur. Microstimulation is a promising way to investigate this issue. In normal monkeys, microstimulation of pontine paramedian reticular formation (PPRF) evokes conjugate, horizontal constant-velocity ramp-like eye movements but, in monkeys with A-pattern strabismus, the same procedure often evoked egregiously disconjugate movements with abnormal vertical components.^9^ Interestingly, however, there were several sites in the monkeys with strabismus at which the evoked movements were horizontal and nearly conjugate (see figure 4D in reference 9 and figures 2E, 2F in reference 20 for examples from exotropic monkey XT1). Because volitional saccades in monkey XT1 were always highly directionally disconjugate, typically by 20 degrees or more,^24^ this indicates that the abnormalities responsible for this animal's A pattern were bypassed for some stimulation sites and not others.

With these results in mind, we reasoned that microstimulation of various nodes along the horizontal and vertical pathways in the brainstem might provide important clues that could help to identify the abnormalities responsible for the cross-axis disconjugacies that characterize pattern strabismus. Because directional disconjugacies occur for both saccades and smooth pursuit,^4^^,^^5^^,^^21^^,^^24^ it makes sense to target structures that are shared by both oculomotor subsystems and that are close to the motor output. The interstitial nucleus of Cajal (INC) is one such structure. Many neurons in this structure show robust bursts of spikes associated with saccades that have a vertical component^25^^,^^26^ and tonic activity related to vertical eye position during fixation, optokinetic nystagmus, and smooth pursuit.^27^^–^^29^ In a recent single-unit recording study, we showed that INC neurons have vertical preferred directions in a normal monkey but, in two monkeys with A-pattern strabismus, many had oblique or even horizontal preferred directions.^13^

If the eye movements evoked by stimulation of INC are vertical and directionally conjugate in monkeys with pattern strabismus, that would indicate that the relevant abnormality lies upstream, or in a parallel pathway. On the other hand, if the evoked movements show directional disconjugacies that are similar to those observed for volitional movements in the same animals, then the crucial abnormality must be very close to the motor output. A third possibility would be that the evoked movements would be approximately normal at some sites and highly directionally disconjugate at other sites (as was the case with PPRF^9^). In a recent modeling paper, we proposed a model (Integrator Cross-talk Model) that assumes that the cross-axis disconjugacies that characterize pattern strabismus result from cross-talk at the level of horizontal and vertical neural integrators in nucleus prepositus hypoglossi (NPH) and INC, respectively. In normal animals, experiments using transneuronal labeling with rabies virus have demonstrated projections from INC to abducens nucleus.^30^^,^^31^ In normal monkeys, this projection is, presumably, too weak to have much of an influence on the direction of eye movements. Indeed, microstimulation of INC in normal monkeys evokes conjugate torsional and vertical movements with little or no horizontal movement.^32^ In our recent modeling paper, we suggested that the functional connectivity between INC and abducens nucleus might be abnormally strong in pattern strabismus.^23^

In that same modeling paper, we also proposed a model that postulates a more general breakdown of directional tuning in pattern strabismus (Distributed Crosstalk Model).^23^ If this is correct, we should find abnormalities of directional tuning in numerous brainstem areas. Microstimulation of any one area in the brainstem should evoke eye movements with directional disconjugacies that would not necessarily match what is observed for volitional movements in the same animal. Thus, these two models make different predictions about the movements that would be expected from stimulating INC. The present study is designed to test these predictions.

## Methods

### Subjects and Surgical Procedures

**Figure 1.**
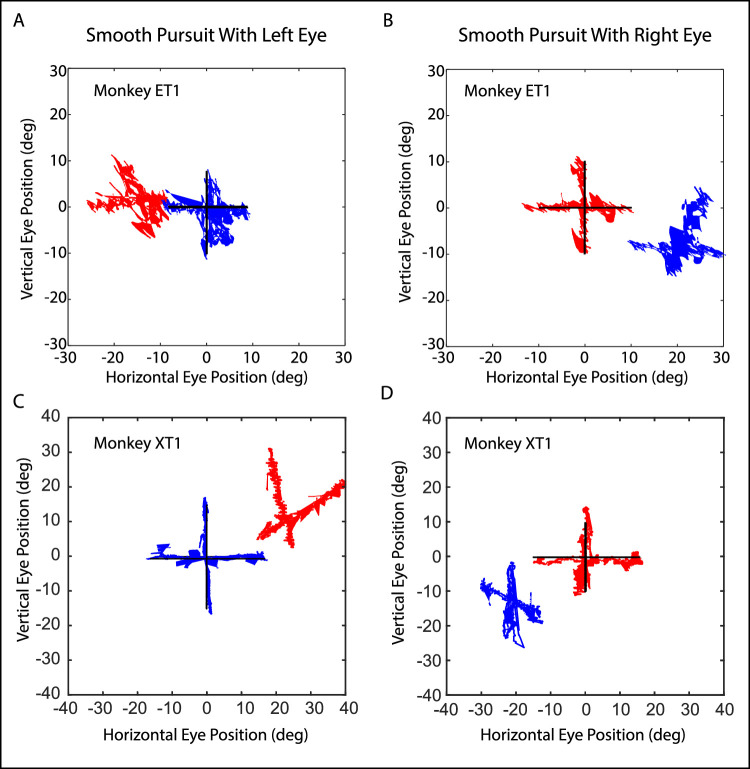
Hess plots for monkeys ET1 (**A,** **B**) and XT1 (**C,** **D**). When the left eye (*left column*) or the right eye (*right column*) tracks the vertically moving target, the fellow eye moves at an oblique angle such that there is a divergent change in the horizontal strabismus angle on downgaze (decreased esotropia for monkey ET1 and increased exotropia for monkey XT1). Conversely, as the viewing eye moved upward, there was a convergent change in the horizontal strabismus angle.

Two macaque monkeys, including one *Macaca mulatta* (Monkey ET1) and one *Macaca nemestrina* (monkey XT1) served as subjects. Monkeys ET1 and XT1 are the same individuals as the similarly named animals in our recently published study of single INC neurons in pattern strabismus.^13^ A permanent esotropia was induced in infancy in monkey ET1 by injecting botulinum toxin into the lateral rectus muscle during the first week of life.^33^^,^^34^ Three additional injections were performed between the ages of 4 months and 2 years. Recording experiments began at 3 years of age, at which time, the monkey had a strong A-pattern esotropia. The horizontal strabismus angle typically ranged from 10 degrees to 25 degrees, mostly depending on vertical eye position. Monkey XT1 had a strong A-pattern exotropia, which resulted from a bilateral medical rectus tenotomy^35^ during the first postnatal week. The horizontal misalignment was typically near 15 degrees on upgaze and 35 degrees on downgaze. Hess plots for both animals can be seen in Figure 1 of our INC single unit recording study.^13^ None of the animals showed a detectable nystagmus during the performance of our behavioral tasks on a typical day.

The Institutional Animal Care and Use Committee at the University of Washington approved the protocol, and all experimental and surgical procedures complied with the ARVO Statement for the Use of Animals in Ophthalmic and Vision Research and the National Institutes of Health Guide for the Care and Use of Laboratory Animals. Eye coils were surgically implanted underneath the conjunctivae of both eyes. This allowed eye position to be measured with high spatial and temporal resolution.^36^^,^^37^ A titanium head post (Crist Instruments Co., Inc., Hagerstown, MD, USA) was implanted on the skull of each animal so that the head could be restrained during the experiments. A 16 mm craniotomy was then performed, with the location chosen to permit access to oculomotor nucleus and INC. Our surgical procedures are described in greater detail in previously published studies.^38^^,^^39^

### Behavioral Tasks and Visual Display

Two behavioral tasks were used. For both, a red laser was used to back-project a 0.25 degree spot onto a tangent screen that was positioned 57 cm from the monkey's eyes. A small amount of applesauce was delivered at regular intervals if at least one of the animal's eyes were within 5 degrees of the target.

The *Target step saccade task* was used while driving the electrode down, and while recording INC neurons for a previously published study.^13^ The location was selected by the computer. Target eccentricities could be 0 degrees, 2 degrees, 4 degrees, 6 degrees, 8 degrees, 10 degrees, 12 degrees, 15 degrees, 18 degrees, and 22 degrees from straight ahead, in any of 8 possible directions. The target always stepped to a new location after fixation intervals ranging from 1.5 to 5 seconds.

The *Fixation task* was used for microstimulation. For this task, the target location was selected by the experimenter, by manually shifting the target in 5 degree horizontal or vertical steps, over a range of ±25 degrees. By combining horizontal and vertical steps, oblique locations could be reached. Microstimulation trains were delivered at unpredictable intervals during the performance of this task.

### Localization of INC and Microstimulation

INC is located just dorsal and lateral to oculomotor nucleus. As a first step, therefore, we located the oculomotor nucleus, which is easily identifiable by the well-known “beehive” sound caused by the high frequency eye position related tonic activity. INC was initially identified by the following neurophysiological criteria: (1) the presence of burst-tonic and tonic neurons that modulate in association with vertical eye position. The latter cell type is rarely found in oculomotor nucleus. (2) The absence of the “beehive” sound. (3) Microstimulation evoked vertical eye movement and the eyes remained near their final locations after the end of each train. In contrast, when the oculomotor nucleus is stimulated, the eyes rapidly return to a position near the animal's volitionally chosen locations following the end of the stimulation train. When targeting the left INC, our angled approach allowed us to continue the track until the electrode passed out of the INC and into the OMN. This was done to help ensure that our stimulation sites were far enough away from the OMN to prevent current from spreading into this structure.

Microstimulation consisted of trains of biphasic pulses, with current levels ranging from 10 µA to 30 µA. Data were collected using stimulation frequencies of 300 and 400 Hz and train durations ranging from 50 to 600 ms. The long train durations were used in an effort to replicate the parameters used in a previous INC stimulation study in normal monkeys.^32^ For all data analyses reported below, however, the train duration was either 50 ms (*n* = 1 site), 100 ms (*n* = 25 sites), or 200 ms (*n* = 10 sites). Train durations of 200 ms were used at sites at which the evoked movements had a relatively low velocity; the goal was to obtain movements with large enough amplitudes to reliably determine the direction.

### Data Analysis

Data acquisition was accomplished using Spike 2 software (Cambridge Electronic Design, Cambridge, UK). All other analyses were conducted using custom scripts and functions, written in Matlab (Mathworks, Natick, MA, USA). Horizontal and vertical strabismus angle were computed for each millisecond of data, using Equation 1:

(1) $$\begin{array}{c} S=P_{left}-P_{right}\; \end{array}$$

where S is the strabismus angle (either horizontal or vertical), P_right_ is the position of the right eye (either horizontal or vertical), and P_left_ is the position of the left eye (either horizontal or vertical). Instantaneous horizontal and vertical eye velocity were estimated using 7-point parabolic differentiation of horizontal and vertical eye position signals, respectively.

Data for a given microstimulation train were excluded if the vectorial velocity of either eye exceeded 6 degrees/second when the first pulse was delivered. If the vertical velocity was less than this threshold, the eye position and velocity data were analyzed within a time window that began 10 ms after the first pulse of the train and ended 10 ms after the final pulse of the train. For stimulation-evoked movements, the onsets of the horizontal and vertical components were detected separately. The onset was taken to be the first point in time at which the component velocity, of at least one eye, exceeded 6 degrees/second and remained above that threshold for at least 10 ms. Separate latency estimates were computed for the horizontal and vertical components of the evoked movements. Latency was defined as the number of milliseconds between the first pulse of the stimulation train and the onset of the horizontal or vertical component. The horizontal and vertical amplitudes of the evoked movements were computed by subtracting the final eye position (i.e. 10 ms after the final pulse) from the initial eye position (i.e. 10 ms after the first pulse).

## Results

Monkeys ET1 and XT1 are the same animals as the similarly named subjects in our recent single unit recording study of INC.^13^ Figure 1 (redrawn from Pallus et al. 2019) shows Hess plots for monkeys ET1 (see Figs. 1A, 1B) and XT1 (see Figs. 1C, 1D), obtained during horizontal and vertical smooth pursuit with the left eye (See Figs. 1A, 1C) and the right eye (see Figs. 1B, 1D) tracking the target.

Microstimulation was delivered to 50 sites, including 14 from monkey ET1 and 36 sites from monkey XT1. Nine of these sites (3 from ET1 and 6 from XT1) were excluded from further analysis due to evidence of current spread into oculomotor nucleus (horizontal and/or vertical eye position showed an exponential back-slide after stimulation offset). Another five sites (4 from ET1 and one from XT1) that were believed to be in INC (based on single unit recording) were excluded from analysis because eye movements were not evoked. Note that this happens for some INC stimulation sites in normal monkeys.^32^

**Figure 2.**
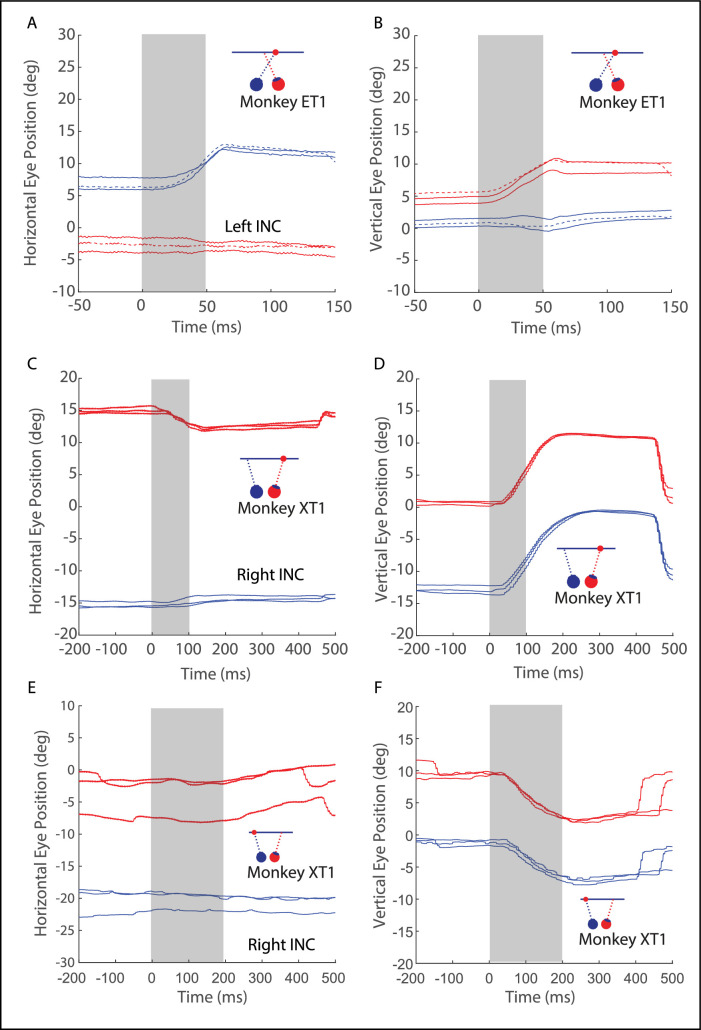
Example data from two microstimulation sites in INC. For each panel, the position of the *red filled circle* in the “eye schematic” indicates which eye the monkey was using to fixate. The *left column* (panels **A**, **C**, and **E**) plots the change in horizontal eye position as a function of time. The *right column* (panels **B**, **D**, and **F**) plots the change in the vertical eye position as a function of time. The *gray shaded area* indicates the period of microstimulation. *Red* = right eye and *blue* = left eye. For the two sites shown in panels **A** through **D**, horizontal movement was consistently observed for one eye but not the other. In addition, for both of these sites, the vertical component of the evoked movements differed for the two eyes. By contrast, the movements evoked from the third site (panels **E**, **F**) were vertical and conjugate. *Dashed lines* in panels **A** and **B** show a trial in which stimulation was delivered while the monkey had looked away from the target. Note that the evoked movement was nearly identical to the two trials for which the monkey was fixating the target with the left eye.

Figure 2 shows data for three example sites, one from monkey ET1 (see Figs. 2A, 2B) and two from monkey XT1 (see Figs. 2C–2F). The change in horizontal (see Figs. 2A, 2C, 2E) and vertical (see Figs. 2B, 2D, 2F) eye position is plotted as a function of time. For two of the sites, the evoked movements were highly directionally disconjugate, with a consistent short-latency horizontal component for one eye but not the other. Note that the eyes remained near their new orbital positions after the end of the microstimulation train, for both the horizontal and vertical components; this indicates that the artificially imposed command was mathematically integrated, which confirms that the evoked movement was due to activation of neurons in INC and not current spread to the oculomotor nucleus.^32^ For the site shown in Figures 2E and 2F, however, the evoked movements were vertical and almost perfectly conjugate. The sites shown in the middle row and the bottom row are from the same animal, yet the evoked movements were normal for one of the sites and highly abnormal for the other.

**Figure 3.**
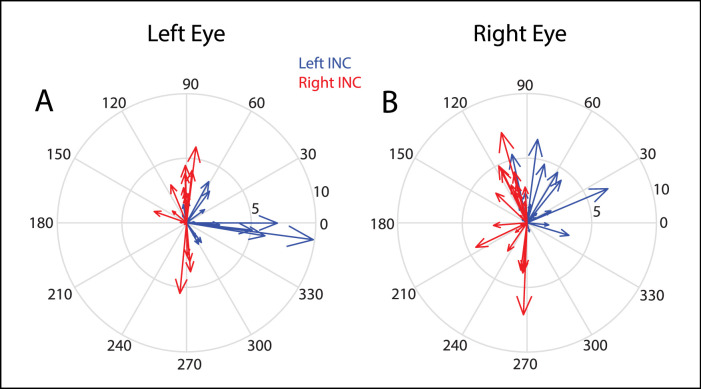
Compass plots showing, for each stimulation site, the mean direction of the evoked movements. *Red* = stimulation sites in the right INC and *blue* = stimulation sites in the left INC. Note that, for some sites, the directional abnormalities were quite egregious, with the movements evoked in one eye being predominantly horizontal. For other sites, the evoked movements were vertical and mostly conjugate.

**Figure 4.**
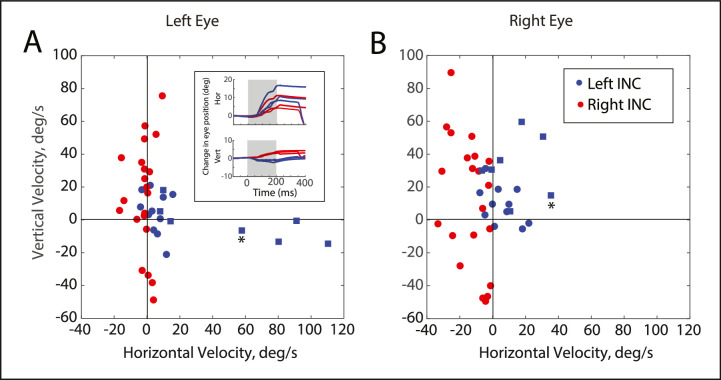
Scatterplots, showing the relationship between the mean vertical and horizontal velocity for each stimulation site. *Blue symbols* indicate sites in the left INC and *red symbols* indicate stimulation sites in the right INC. *Squares* represent sites from monkey ET1; and the *circles* represent sites from monkey XT1. For stimulation sites in the left INC, there was a tendency for the evoked movements to show a rightward component, often for both eyes. For the right INC, the horizontal component was typically very small for the left eye but was usually leftward for the right eye. The inset is an example site at which the evoked movements were predominantly rightward for both eyes. Note that the eyes remain at the new locations following the end of stimulation, which indicates that the horizontal movement was not due to current spread into the medial rectus subdivision of the oculomotor nucleus.

This high degree of site-to-site variability was a common feature in our data set. Figure 3 shows the mean directions and amplitudes of the evoked movements for each stimulation site. Bearing in mind that INC stimulation in normal monkeys evokes conjugate vertical movements with very little horizontal movement,^32^ the directions of the evoked movements in the monkeys with A-pattern strabismus were highly abnormal for some sites and not for others. For sites in the left INC (blue arrows), in particular, the directional disconjugacy was often egregious, a fact that can be inferred from a close examination of this figure; the vertical component for the left eye could be either upward (6/18 sites) or downward (7/18 sites) but the right eye showed an upward component for 15 of 18 sites. In addition, for some sites, the horizontal component was larger than the vertical component for one or both eyes (see Figs. 2A, 2B for an example). For sites in the right INC (red arrows), the left eye's movement was within 30 degrees of vertical for 18 of 21 sites and the direction of the left eye's movement deviated from vertical by more than 10 degrees for only 5 sites. For the right eye, a clear leftward component was observed for most sites in the right INC, although it was usually smaller than the vertical component. For 7 of 21 of these sites, however, the direction of the right eye's movement deviated from vertical by >30 degrees. For some sites, particularly in the left INC, the left eye moved downward while the right eye moved upward. This phenomenon was observed in both monkeys. These tended to be sites from which large horizontal movements were evoked for the left eye (the inset in Fig. 4A shows 3 stimulation trials from one such site). For this site, please note, in particular, that the horizontal component was considerably larger than the vertical component for both eyes, and both eyes remained near their final horizontal positions after the end of stimulation. It is quite striking to see such movements evoked by microstimulation of a premotor site that directly drives vertical rectus motoneurons.

### Horizontal Component

Figure 4 plots the mean vertical velocity as a function of the mean horizontal velocity, for each eye for each site. Plotted in this way it is easy to see that stimulation of most sites in the right INC evoked nearly normal movements in the left eye (red dots in Fig. 4A); there was little or no horizontal movement for most sites. It is also clear that, for these same sites, the right eye typically showed a clear leftward component (red dots in Fig. 4B). One can also see that the movements evoked by stimulation of the left INC (blue dots) were clearly more likely to be upward for the right eye than for the left eye. Finally, stimulation of the right INC was more likely to evoke movements with a leftward component for the ipsilateral eye, whereas stimulation of the left INC typically evoked movements with a rightward component for the ipsilateral eye. There was no significant difference between the mean horizontal velocity for the left versus the right eyes, for stimulation sites in the left INC (two-tailed *t*-test, *P* = 0.09). However, for sites in the right INC, the mean horizontal velocity for the right eye was significantly more negative (leftward) for the right eye, compared to the left eye (*P* < 0.0001). Moreover, the mean horizontal velocity differed significantly for the right INC versus the left INC for both eyes (*P* < 0.01 for both comparisons). For these comparisons, the alpha level was corrected to 0.0125 using the Bonferroni method.

For monkey ET1, there were four sites (all in left INC) at which the left eye's horizontal velocity exceeded 50 degrees/second (see blue squares in Fig. 4A). At each of these sites, the horizontal velocity for the right eye was notably lower. One might wonder whether this odd result might be due to current spread into the medial rectus subdivision of the oculomotor nucleus. However, as one can see from the inset in Figure 4, despite the large horizontal component for the left eye, the eyes remained near their new locations after stimulation offset. In addition, the oculomotor nucleus was encountered on each of these tracks, at a depth of at least 1 mm below the stimulation site. Thus, we think current spread into the oculomotor nucleus is an unlikely explanation for the horizontal component of the evoked movements. In any event, one can see from Figure 4 that the 4 sites at which the most egregiously abnormal movements were evoked all came from monkey ET1. Across the entire data set, the mean absolute value of the horizontal velocity was 12.5 degrees/second for the left eye and 13.7 degrees/second for the right eye.

There were no consistent relationships between the vertical and horizontal components. For the right eye, for stimulation sites in the right INC, horizontal components (when present) were always leftward; this was true regardless of whether the vertical component was upward or downward (see Fig. 4B). Of the four possible relationships (left eye-left INC, left eye-right INC, right eye-left INC, and right eye-right INC), the slope of the linear regression line (fit to the relationship between horizontal and vertical velocity) differed significantly from 0 only for left eye-left INC (*P* = 0.02). Even for that condition, however, the horizontal and vertical components did not consistently follow what one would expect for monkeys with A patterns; both upward and downward movement tended to be associated with rightward horizontal components (see the blue dots in Fig. 4A).

Despite the large site-to-site variability, a close examination of Figure 4 shows that the larger horizontal movements tended to take the form of adduction of the eye ipsilateral to the stimulation site.

### Directional Disconjugacy

**Figure 5.**
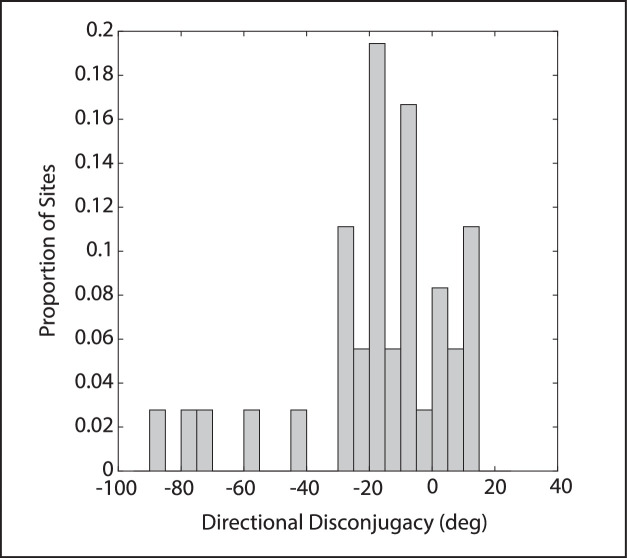
Histogram showing the distribution of directional disconjugacies across stimulation sites. Note the large variability. For some sites, the evoked movements for both eyes were in almost identical directions. For other sites, the directional disconjugacy was quite egregious (>40 degrees).

The above analyses indicate that microstimulation of some sites in INC in monkeys with A-pattern strabismus evokes movements with abnormally large horizontal components. We now report the results of quantifying the directional disconjugacy of these evoked movements. For monkey ET1, the mean direction of evoked movements was significantly different for the two eyes for six of seven sites (2-tailed *t*-tests); for all sites the right eye's direction was deviated in a counter-clockwise direction, relative to the left eye's direction. The directional disconjugacy tended to be quite egregious (mean = −52.1 degrees). For monkey XT1, the direction of the evoked movements differed significantly for the two eyes for 13 of 29 sites. The right eye's direction was deviated in a counter-clockwise direction for 11 of these 13 sites and in a clockwise direction for 2 sites (mean = −9.0 degrees). Figure 5 shows a histogram depicting the distribution of directional disconjugacies for our stimulation sites. It is important to note the large site-to-site variability. Indeed, the directional disconjugacy was less than 10 degrees for 12 sites and it was less than 5 degrees for 4 sites. At the other extreme, there were 5 sites for which the disconjugacy exceeded 40 degrees. For monkey XT1, for example, the directional disconjugacies ranged from 1.5 degrees to −40.6 degrees. Furthermore, for the site with the directional disconjugacy of 1.5 degrees, the deviation from straight upward was less than 5 degrees for both eyes. Thus, at this site, the evoked movements were entirely normal.

### Amplitude Disconjugacy

Several studies have reported that the two eyes often make saccades of different amplitudes in the strabismus.^21^^,^^24^^,^^40^ To disambiguate amplitude and directional disconjugacy, it is necessary to consider the vectorial amplitude of eye movements.^24^ With this in mind, we used two-tailed *t*-tests to compare the vectorial amplitudes of stimulation-evoked movements for each site. For monkey ET1, the vectorial amplitudes differed significantly for the two eyes for five of seven sites. In each case, the amplitude was larger in the left eye (mean = left eye = 9.3 degrees; and the right eye = 5.9 degrees). For monkey XT1, the vectorial amplitudes differed significantly for the 2 eyes for 12 of 29 sites. In each case, the amplitude was larger in the right eye (mean = left eye = 4.9 degrees; and the right eye = 5.8 degrees).

### Effect of Initial Eye Position

A potential limitation of the above analyses is the fact that, because our monkeys had strabismus, the eyes were always in different orbital positions at the time of stimulation onset. If there is any relationship between initial eye position and the direction and/or velocity of the evoked movements, then disconjugacy might be attributable solely to the fact that the eyes always began in different orbital positions. According to this hypothesis, the disconjugacy of evoked movements should disappear if one controls for initial eye position.

To address this possibility, we plotted the relationship between initial eye position and the amplitudes of evoked movements for each eye, using Matlab's curve fit tool. This procedure yielded 95% confidence intervals for both the y-intercept and the slope, for both vertical and horizontal amplitudes. Note that, for this analysis to be meaningful, the range of amplitudes of the evoked movements must be large enough to detect a correlation between initial eye position and the amplitude of the evoked movement. With this in mind, a given site was included in this analysis only if the range of vectorial amplitudes was at least 8 degrees. This threshold was chosen with the goal of achieving an optimal compromise between the need to include as many stimulation sites as possible and the need to exclude sites at which a correlation between amplitude and initial eye position would be undetectable.

**Figure 6.**
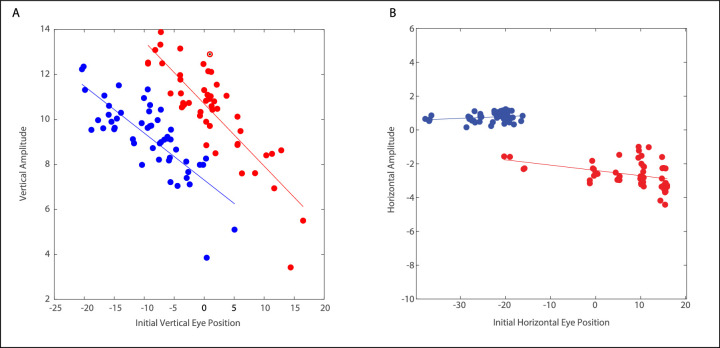
Relationship between initial eye position and the component amplitude of the evoked movement for one example site, from the right INC in monkey XT1. *Blue* = the left eye and *red* = the right eye. (**A**) There was a strong relationship between vertical amplitude and initial vertical eye position. However, for any given vertical eye position, the vertical amplitudes were larger in the right eye. (**B**) The relationship between horizontal amplitude and initial horizontal position was weak or non-existent. Note that the horizontal component was always rightward for the left eye and leftward for the right eye.

Figure 6 shows examples of these relationships for both the horizontal and vertical components for one example site. For both eyes, there is a clear relationship between the initial vertical eye position and the vertical amplitude of the evoked movements (see Fig. 6A) but it is also clear that this cannot account for the vertical disconjugacy. For any given initial vertical eye position, the vertical amplitude was consistently greater for the right eye. For the horizontal component, the correlation between the initial eye position and the horizontal amplitude was weak or nonexistent (see Fig. 6B).

For the vertical component for the eye ipsilateral to the stimulation site, the slope of the component amplitude versus the IEP relationship was significantly different from 0 (i.e. the 95% confidence interval did not include 0) for 15 of 18 sites. For the vertical component for the contralateral eye, the slope was significantly different from 0 for 15 of 17 sites. For the horizontal component for the ipsilateral eye, the slope was significantly different from 0 for 10 of 18 sites. For the horizontal component for the contralateral eye, the slope was significantly different from 0 for 9 of 17 sites. Although the slope of the relationship between the horizontal initial eye position and the amplitude of the horizontal component often differed significantly from zero, it was consistently small for both eyes (-0.05 and -0.06) for the left eyes and right eyes, respectively.

To compare the y-intercepts for the 2 eyes, a given site was included only if the range of amplitudes of both eyes was at least 8 degrees (*n* = 17). For the vertical component of the evoked movements, the y-intercepts for the 2 eyes were significantly different for 15 of 17 sites. For all 15 of these sites, the vertical components of the evoked movements were more upward for the right eye. For six of these sites, this meant that the movements evoked when the vertical eye position was 0 would be upward for the right eye but downward for the left eye (at the same vertical eye position). For 8 of 15 of these sites, the left INC was stimulated and for 7 of 15 of these sites the right INC was stimulated. For horizontal amplitude, the y-intercepts for the 2 eyes were significantly different for 15 of 17 sites. It is noteworthy, however, that the y-intercepts were quite small for horizontal amplitude for the majority of sites, indicating that the horizontal amplitudes tended to be small for both eyes when the initial horizontal eye position was 0. Contrast this with the relatively large horizontal movements observed for some sites (see Figs. 2, 3) and some of the solid blue dots in Figure 4. This suggests that the larger horizontal movements tend to occur when the initial vertical eye position is either upward or downward. Thus, for both the horizontal and vertical components of the evoked movements, the amplitude disconjugacy is not solely a consequence of the fact that the eyes begin in different orbital positions.

### Relationship Between Change in Horizontal Strabismus Angle and Vertical Amplitude

**Figure 7.**
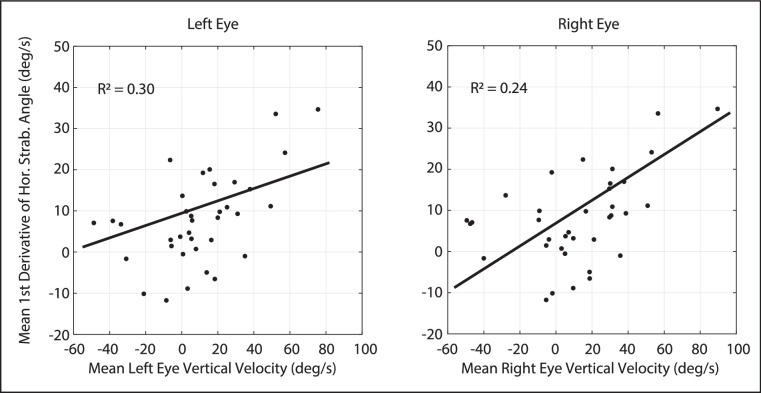
Relationship between the mean first derivative of horizontal strabismus angle (mathematically equivalent to vergence velocity) and the mean vertical velocity, for all stimulation sites. For both eyes, there is a significant positive correlation, which is what one would expect for monkeys with A-pattern strabismus. Nonetheless, there were some sites at which the mean first derivative of horizontal strabismus angle was the opposite of what one would expect for a monkey with an A pattern, given the direction of the mean vertical amplitude.

As noted in the introduction, the Integrator Crosstalk Model^23^ predicts that, for movements evoked by microstimulation of the INC, the relationship between the vertical velocity and the first derivative of the horizontal strabismus angle (mathematically, but not physiologically, equivalent to vergence velocity) should be similar to what is observed for the volitional movements. If the abnormality responsible for these animals’ A-patterns is at, or downstream from, the level of INC, then one might expect upward movement to be consistently associated with convergent changes in horizontal strabismus angle and downward movement to be consistently associated with divergent changes in horizontal strabismus angle. Figure 7 shows this relationship for our 2 monkeys with A-pattern strabismus. Each data point compares the mean vertical velocity and the first derivative of the horizontal strabismus angle for a single stimulation site. For both eyes, there was a weak, but statistically significant, positive correlation between the vertical velocity and the first derivative of the horizontal strabismus angle (left eye: R^2^ = 0.30, slope = 0.22 [95% confidence bounds = 0.10, 0.34); and the right eye: R^2^ = 0.24, slope = 0.17 [95% confidence bounds = 0.06, 0.28]). Nonetheless, there were five sites for which upward movement of the right eye was associated with a divergent change in the horizontal strabismus angle, and nine at which downward movement was accompanied by a convergent change in horizontal strabismus angle. This is, of course, the opposite of what one would expect for a monkey with an A pattern.

**Figure 8.**
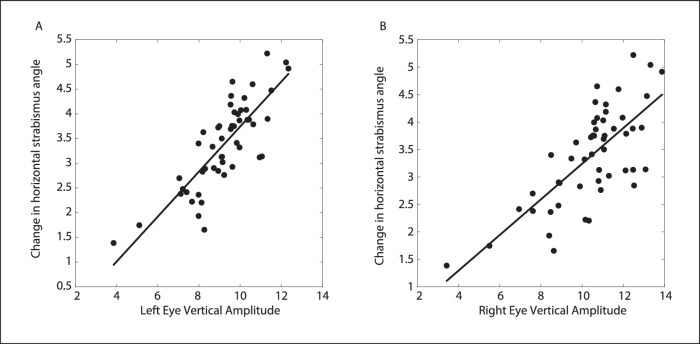
The relationship between the change in horizontal strabismus angle and vertical amplitude, for one example site. Note that, for both eyes, larger upward components were associated with more convergent changes in the horizontal strabismus angle.

In Figure 7, each data point represents the mean values for a single stimulation site. Because, as we have seen, the amplitude of the vertical component varied with initial vertical eye position for many sites, a plot based on mean values may obscure some of the complexity of these relationships. With this in mind, we also sought to determine whether, for each site, vertical amplitude was significantly correlated with the change in horizontal strabismus angle. As before, any site with a range of vertical amplitudes less than 8 degrees was excluded from this analysis. Figure 8 shows data from one example site in monkey XT1. Stimulation of this site, in the right INC, consistently evoked movements with an upward component in both eyes (with a slight leftward component in the right eye). For both eyes, the larger the upward component the greater was the decrease in the horizontal strabismus angle; for an animal with exotropia, this is mathematically equivalent to convergence and it was consistent with this animal's A pattern.

Overall, a significant positive correlation between the vertical amplitude of the ipsilateral eye and the change in horizontal strabismus angle (i.e. convergent changes had positive values) was found for 12 of 18 sites; a significant negative correlation (which is the opposite of what would be expected for a monkey with an A pattern) was found for 4 of 18 sites. However, when this correlational analysis was based on the vertical amplitude of the contralateral eye, a significant positive correlation was found for all 17 of the sites at which the range of vertical amplitudes was at least 8 degrees.

The data shown in Figures 7 and 8 may seem somewhat contradictory, with the former being inconsistent with the predictions of the Integrator Crosstalk Model, and the latter consistent with it. The explanation is that, for some sites, stimulation consistently evoked movements with an upward component and divergent changes in the horizontal strabismus angle, yet larger (i.e. more upward) movements were less divergent than smaller (less upward) movements. Similarly, for some sites, stimulation evoked downward movements that consistently involved a convergent change in horizontal strabismus angle, but larger (more downward) movements were less convergent. Thus, for a minority of sites, the correlation was in the expected direction for an A pattern but the mean change in horizontal strabismus angle was the opposite from what would be expected for an animal with an A pattern.

Next, we asked whether the horizontal component of the evoked movements (when present) differed depending on which eye was fixating the target. This analysis was only performed for a particular eye and stimulation site if the mean absolute value of the horizontal component was at least 1 degree (for at least one fixating eye condition). For the left eye, the horizontal component was significantly larger if that eye was fixating on the target for 9 of 13 sites and significantly smaller when that eye was fixating for 2 of 13 sites. For the right eye, the horizontal component was significantly larger if that eye was fixating the target for 3 of 21 sites and significantly smaller for 10 of 21 sites. As one can see from Figure 4, the larger horizontal movements tended to take the form of adduction of the eye ipsilateral to the stimulation site, regardless of which eye was fixating on the target.

## Discussion

The Integrator Crosstalk Model predicts that microstimulation of INC in a monkey with A-pattern strabismus should evoke directionally disconjugate eye movements that should result in convergent changes in the horizontal strabismus angle when the vertical component is upward and divergent changes when it is downward. This prediction arises from the fact that this model places the neural basis for pattern strabismus, and directional saccade disconjugacy, at the level of the neural integrators in the INC and NPH. Specifically, this model attributes the relationship between horizontal strabismus angle and vertical eye position to an inappropriately strong functional connectivity from the INC to the NPH.

Microstimulation of the INC generated vertical eye movements in monkeys with strabismus but, for some sites, the pattern of activation was quite dissimilar to that which occurs in association with volitional saccades.^32^ Interpretation of this observation is somewhat complicated by the fact that microstimulation creates a pattern of activation that is quite dissimilar to that which occurs during natural behavior. In the INC, neurons with upward and downward preferred directions are intermixed.^41^ For naturally occurring eye movements, tonic and burst-tonic neurons increase their firing rates for eye movements that have on-direction vertical components and decrease their firing rates for those that have off-direction vertical components.^41^ Microstimulation of this structure, by contrast, activates all neurons near the electrode tip, regardless of their preferred directions. It is believed that the direction of the eye movement resulting from INC stimulation represents the net output of these competing signals.^32^

The Integrator Crosstalk Model predicts that, for a monkey with A-pattern strabismus, this net output drives neurons in abducens nucleus (a connection that is has been demonstrated in normal animals^30^^,^^31^; see Figs. 1C, 1G in Walton and Mustari^23^) to an abnormally strong degree. Because the abnormally strong cross-talk lies in the downstream connections from the INC, the model predicts a straightforward and consistent relationship between the vertical amplitudes of evoked movements and the change in horizontal strabismus angle. Furthermore, the directional abnormalities for some sites were far more egregious than anything we observed for volitional eye movements in the same animals (see Figs. 2A, 2B, Fig. 3, Fig. 4A). For other sites, stimulation evoked movements that were vertical and nearly conjugate. In these respects, the present data seem inconsistent with this model.

On the other hand, for all stimulation sites at which this analysis was performed, the change in horizontal strabismus angle was positively correlated with the vertical amplitude of the contralateral eye. This implies that there is at least some abnormal vertical-to-horizontal crosstalk at the level of, or downstream from, the INC. We suggest that this pattern of results is most consistent with the Distributed Crosstalk Model, which also uses an abnormally strong INC-to-abducens connection but only as a part of a more general breakdown of directional tuning throughout the saccadic system. Thus, this model more easily accounts for somewhat “messy” data, such as what we see in Figure 4 of the present paper.

Here, it is worth discussing the work of Demer and colleagues. Some of these studies have provided compelling evidence that the eye muscle pulleys can be mislocated in a way that can contribute to the abnormalities that characterize pattern strabismus.^42^^–^^44^ In another study, they showed that, in humans with concomitant esotropia, the medial rectus muscle is of supranormal size.^45^ Thus, although the present results provide compelling additional evidence for neurophysiological abnormalities in pattern strabismus, this does not exclude the role of peripheral abnormalities.

We found a very similar idiosyncratic variability of disconjugacies between sites for microstimulation of PPRF in monkeys with pattern strabismus.^9^ The movements evoked by stimulation of some PPRF sites were conjugate and horizontal, whereas stimulation of other sites evoked movements that were far more directionally disconjugate than volitional saccades in the same animal. This implies a “patchwork” aspect to the abnormalities; the most parsimonious explanation for this result is that signals from some parts of PPRF are appropriately routed only to downstream areas involved in horizontal eye movement, whereas those from other parts of the same structure are also sent to downstream areas that play a role in the generation of vertical movements. Similar reasoning could be applied to the results of the present study. Thus, the results of these two microstimulation studies suggest the possibility that abnormal cross-talk between horizontal and vertical pathways may occur at multiple levels. Additional work will be needed to determine if this is the correct interpretation, however.

As one can see from Figure 4, the larger horizontal components tended to occur in the eye ipsilateral to the stimulation site. This was true regardless of which eye was being used to fixate on the target. The raises the question of how, in these monkeys with pattern strabismus, signals from some (but not all) sites in the INC could have found their way to the medial rectus motoneurons. As discussed in our previously published modeling paper,^23^ there is a potential pathway from the INC to the abducens to medial rectus motoneurons but, for sites at which horizontal movement is limited to abduction of the ipsilateral eye, this would require selective activation of abducens internuclear neurons. Whereas this remains to be a possibility, an alternative possibility would be direct projection from the INC to the oculomotor nucleus. In normal monkeys, of course, this projection is limited to vertically acting motoneurons but that might not be the case in pattern strabismus. It is well known that there are numerous connections in infant mammals that are selectively lost during early postnatal life.^46^^–^^48^ Perhaps, at birth, INC neurons drive medial rectus motoneurons and these connections are functionally lost in normally developing primates. According to this idea, this developmental pruning process would be only partially successful in pattern strabismus, leaving inappropriate functional connections between the vertical neural integrator and medial rectus motoneurons.

The preferred directions of individual neurons in the PPRF^10^ and INC^13^ were also found to be abnormal in pattern strabismus but, once again, it was difficult to establish a clear relationship between the neurophysiological abnormalities of a particular structure and the cross-axis disconjugacies that characterize pattern strabismus. These results, too, seem to conform to the predictions of the Distributed Crosstalk Model. Taken together, these studies support the view that pattern strabismus is a complex phenomenon that is associated with both peripheral abnormalities and neurophysiological abnormalities at various levels in the brain.

### Alternative Explanations and Potential Methodological Concerns

As noted in the Methods section, monkey XT1 underwent eye muscle surgery during the first week of life. This leaves open the possibility that the eye muscles and/or orbital tissues in this animal may not have been normal. One might wonder, therefore, whether the disconjugacy of evoked movements, and the abnormal horizontal movement observed for some sites, might be merely a consequence of peripheral factors. Of course, eye muscle surgery has been used to experimentally induce strabismus for decades, and this possibility has been discussed in many of these previous studies (for 2 recent reviews that discuss this issue at length, see Refs. 19 and 20). Briefly, however, note that the directions of evoked movements in this animal varied widely from one stimulation site to another, even for sites on the same side of the brain. In particular, the example site shown in Figures 2A and 2B is from a monkey that did not undergo medial rectus tenotomy and the abnormalities of the evoked movements are egregious; microstimulation of the *vertical* neural integrator caused the left eye to move almost purely rightward. As mentioned above, in our earlier PPRF stimulation paper,^9^ we noted that the evoked movements were conjugate and horizontal for some sites, and egregiously abnormal for other sites on the same side of the brain in the same monkey. Of course, the pulling directions of eye muscles are not going to change merely because the electrode is in a slightly different location within the same brain region. Thus, in both the PPRF study and in the present study, the large site-to-site variability rules out the possibility that the abnormalities of evoked movements are due solely to peripheral factors.

A related point is that, in a normal animal, microstimulation of the INC would not be expected to activate the medial rectus muscle at all, so the issue of whether this muscle has an abnormal pulling direction in monkey XT1 would be moot if the INC were normal.

Additionally, in recent years, we have shown directional abnormalities in both microstimulation and recording studies in several brainstem regions^9^^–^^11^^,^^13^^,^^49^^,^^50^ and the present results are consistent with these previously published studies. Finally, similar abnormalities are observed in monkeys that did not undergo eye muscle surgery. Thus, although it remains possible that the present results might have been affected by peripheral abnormalities in monkey XT1, any such effects cannot entirely explain the data.

Another potential source of concern is the fact that two different methods were used to generate strabismus in this study. This issue is also addressed in our recent review,^20^ and we would refer the concerned reader to this paper. Briefly, the available literature indicates that prolonged deprivation of binocular vision during a sensitive period in early postnatal life – regardless of the cause – leads to a permanent loss of binocular visual responses in visual cortical areas.^51^^–^^56^ This is thought to lead to a “cascade of abnormalities” that ultimately affects the developmental trajectories of brain areas involved in eye movements. Indeed, in our previous studies, we have consistently found that the results were very similar between an animal that underwent eye muscle surgery in infancy and those for which strabismus was induced using other procedures (such as wearing prism goggles).^9^^,^^11^^–^^13^^,^^24^^,^^49^^,^^50^

The INC plays an important role in torsion as well as vertical eye movement.^32^ Pattern strabismus is often described, and treated, as overaction or underaction of oblique muscles. One motivation for this has been the observation that this disorder is often associated with abnormal torsion.^1^ In the present study, we were not able to measure torsion. Thus, it remains unknown whether microstimulation of the INC induces abnormal torsional movement in monkeys with experimentally induced pattern strabismus. However, behavioral studies have shown that torsional abnormalities are not always present in humans with pattern strabismus^57^ and, when they are, the severity of the torsional abnormality is not correlated with the magnitude of the cross-axis disconjugacies^21^ (see our recent review paper for a more thorough discussion of this issue^20^). Given this partial dissociation of these two abnormalities, useful inferences can be made about horizontal-vertical crosstalk in the brainstem, without resolving the question of how INC stimulation affects torsion in monkeys with pattern strabismus.

### Concluding Observations

The present results indicate that, in monkeys with pattern strabismus, there are significant abnormalities in the brainstem that affect the directions of eye movements in ways that differ for the two eyes. For some sites, evoked movements were nearly conjugate, whereas for other sites, the directional disconjugacy was much more egregious than observed for volitional saccades in the same animal. This site-to-site variability rules out the hypothesis that the abnormalities responsible for pattern strabismus are entirely peripheral. More work will be needed to identify the specific neural abnormalities but the present study provides useful constraints that point to brain areas that should be targeted by future work.
